# Dapagliflozin and Anemia Outcomes: A Systematic Review and Meta-Analysis of Effects on Hemoglobin Levels, Anemia Correction, and Incidence in Patients With and Without Heart Failure

**DOI:** 10.7759/cureus.95004

**Published:** 2025-10-20

**Authors:** Huzaifa Rehman, Anastasia Postoev, Anurag Rawat, Mandeep Kaur, Fikadu Woreta, Sonalben Chaudhary, Mohammed Qasim Rauf, Areeba Khan

**Affiliations:** 1 General Medicine, Avicenna Medical College, Lahore, PAK; 2 Opthalmology, Rochdale Infirmary Hospital, Rochdale, GBR; 3 Internal Medicine, Caribbean Medical University, Willemstad, CUW; 4 Interventional Cardiology, Himalayan Institute of Medical Sciences, Dehradun, IND; 5 Hospital Medicine, Hospital Corporation of America (HCA) Florida Capital Hospital, Tallahassee, USA; 6 Internal Medicine, Inova, Silver Spring, USA; 7 Internal Medicine, Zydus Sitapur Hospital, Sitapur, IND; 8 Trauma and Orthopaedics, The Hillingdon Hospitals National Health Service (NHS) Foundation Trust, London, GBR; 9 Critical Care Medicine, United Medical and Dental College, Karachi, PAK

**Keywords:** anemia, dapagliflozin, heart failure, hemoglobin, sglt2 inhibitors

## Abstract

This systematic review and meta-analysis evaluated the impact of dapagliflozin on anemia outcomes across different patient populations. A comprehensive literature search was conducted across multiple databases from inception to September 2025, identifying studies that reported anemia-related outcomes in adult patients receiving dapagliflozin treatment. Seven studies comprising 15,540 participants were included, encompassing randomized controlled trials and observational studies conducted across diverse geographical regions. All studies evaluated 10 mg of dapagliflozin once daily with a minimum four-week follow-up duration. The pooled analysis demonstrated significant improvements in hemoglobin levels with dapagliflozin compared to control groups (standardized mean difference [SMD]: 0.62; 95% CI: 0.43-0.80). Dapagliflozin treatment was associated with an 83% higher likelihood of anemia correction (relative risk: 1.83; 95% CI: 1.47-2.26) and a 78% reduction in anemia incidence (relative risk: 0.22; 95% CI: 0.08-0.60). Subgroup analyses revealed consistent benefits across all studied populations, with the most pronounced effects observed in heart failure patients (SMD: 0.78; 95% CI: 0.44-1.12), followed by chronic kidney disease (0.66; 95% CI: 0.36-0.95) and diabetes patients (0.45; 95% CI: 0.38-0.51). These findings suggest that dapagliflozin's therapeutic benefits extend beyond cardiovascular and renal protection to include meaningful improvements in anemia outcomes across multiple chronic disease states, potentially reducing healthcare burden and improving patient quality of life.

## Introduction and background

Heart failure (HF) affects more than 26 million people worldwide and represents a major public health burden with substantial morbidity and mortality [1]. Anemia, defined as hemoglobin levels below 12.0 g/dL in women and 13.0 g/dL in men, is a frequent comorbidity in HF patients, affecting approximately 15-30% of individuals with chronic heart failure and up to 50% of those with advanced disease [2,3]. The presence of anemia in HF is associated with worse functional status, increased hospitalization rates, and elevated mortality risk, making it an important therapeutic target [4].

Sodium-glucose cotransporter-2 (SGLT2) inhibitors, initially developed for type 2 diabetes management, have demonstrated remarkable cardiovascular and renal benefits beyond glycemic control [5]. Large randomized controlled trials, including DAPA-HF and DELIVER, have established that dapagliflozin significantly reduces the risk of cardiovascular death and heart failure hospitalizations in patients with heart failure across the spectrum of left ventricular ejection fraction [6,7]. These benefits extend to both diabetic and non-diabetic patients, suggesting mechanisms beyond glucose lowering.

Emerging evidence indicates that sodium-glucose cotransporter 2 (SGLT2) inhibitors may have beneficial effects on hematological parameters. Recent systematic reviews and meta-analyses have demonstrated that SGLT2 inhibitors significantly increase hemoglobin and hematocrit levels in patients with type 2 diabetes and chronic kidney disease [8,9]. Post-hoc analyses of the dapagliflozin and prevention of adverse outcomes in heart failure (DAPA-HF) trial revealed that dapagliflozin corrected anemia in 62.2% of patients compared to 41.1% in the placebo group, suggesting clinically meaningful hematopoietic effects [10].

The mechanisms underlying SGLT2 inhibitor-induced improvements in anemia are multifactorial. Proposed mechanisms include enhanced erythropoietin production through activation of hypoxia-inducible factor-2α (HIF-2α), improved tissue oxygenation, hepcidin inhibition leading to increased iron bioavailability, and volume contraction effects [11,12]. Additionally, SGLT2 inhibitors may ameliorate chronic inflammation and oxidative stress, both of which contribute to anemia of chronic disease commonly observed in heart failure patients [13].

Recent clinical studies have shown that dapagliflozin significantly improved glycemic control and alleviated anemia in heart failure patients when used in conjunction with standard treatment, with improvements observed in hemoglobin, hematocrit, and iron metabolism parameters [14]. However, the evidence remains heterogeneous, and the magnitude of benefit may vary across different patient populations and clinical contexts.

Despite growing interest in the hematopoietic effects of SGLT2 inhibitors, no comprehensive systematic review has specifically evaluated the impact of dapagliflozin on anemia outcomes across different patient populations, particularly comparing effects in patients with and without heart failure. Understanding these effects is crucial for optimizing therapeutic strategies and potentially expanding the clinical utility of dapagliflozin beyond its established cardiovascular and renal indications.

## Review

Methodology

This systematic review and meta-analysis were conducted in accordance with the Preferred Reporting Items for Systematic Reviews and Meta-Analyses (PRISMA) guidelines [15].

Literature Search and Search Strategy

A comprehensive systematic literature search was performed across multiple electronic databases, including PubMed/MEDLINE, Embase, Cochrane Central Register of Controlled Trials (CENTRAL), Web of Science, and Clinical Trials from database inception to 1 September 2025. The search utilized both Medical Subject Headings (MeSH) terms and free-text keywords. The primary search terms included: ("dapagliflozin" OR "Forxiga" OR "Farxiga") AND ("anemia" OR "anaemia" OR "hemoglobin" OR "haemoglobin" OR "hematocrit" OR "haematocrit" OR "red blood cell*" OR "erythrocyte*" OR "hematopoiesis" OR "haematopoiesis") AND ("heart failure" OR "cardiac failure" OR "HF" OR "HFrEF" OR "HFpEF" OR "cardiomyopathy" OR "left ventricular dysfunction"). Additional search terms included combinations with "SGLT2 inhibitor" OR "sodium glucose cotransporter 2" to capture broader literature. The search was adapted for each database using appropriate syntax and controlled vocabulary. Reference lists of included studies, relevant systematic reviews, and meta-analyses were manually searched to identify additional eligible studies. Conference abstracts from major cardiology and endocrinology meetings were also reviewed. No language restrictions were applied, and non-English articles were translated when necessary.

Study Selection

Two independent reviewers screened all retrieved records using a standardized two-stage process. Initial screening involved title and abstract review, followed by full-text assessment of potentially eligible studies. Disagreements were resolved through discussion with a third reviewer. Inclusion criteria comprised randomized controlled trials (RCTs) and observational cohort studies; adult patients (≥18 years) receiving dapagliflozin treatment; studies reporting anemia-related outcomes (hemoglobin levels or anemia correction rates); comparison with placebo, standard care, or other antidiabetic medications; minimum follow-up duration of four weeks; and available data on baseline and post-treatment hematological parameters. Exclusion criteria included case reports, case series, editorials, and review articles; studies with insufficient data on anemia outcomes; duplicate publications (the most recent or comprehensive report was included); studies that included pediatric populations; and conference abstracts without sufficient methodological detail or outcome data.

Data Extraction

Data extraction was performed independently by two reviewers using a standardized, pre-piloted data extraction form. The following information was systematically extracted: study characteristics, including first author, year of publication, study design, geographic location, and sample size; population characteristics, encompassing age, sex distribution, and baseline comorbidities (diabetes mellitus, chronic kidney disease, and heart failure type and severity); intervention details, covering dapagliflozin dosage, administration frequency, treatment duration, and comparator group details; and outcome measures.

Quality Assessment

Quality assessment was conducted independently by two reviewers using appropriate tools based on the study design. For randomized controlled trials, the Cochrane Risk of Bias tool version 2 (RoB 2) was employed [16], evaluating five domains: bias arising from the randomization process, bias due to deviations from intended interventions, bias due to missing outcome data, bias in measurement of the outcome, and bias in selection of the reported result. Each domain was rated as "low risk," "some concerns," or "high risk," with an overall risk of bias judgment assigned to each trial. For cohort studies, the Newcastle-Ottawa Scale (NOS) was utilized [17], assessing three main categories: selection of study groups (maximum 4 stars), comparability of groups (maximum 2 stars), and assessment of exposure/outcome (maximum 3 stars). Studies scoring ≥7 stars were considered high quality, 5-6 stars moderate quality, and <5 stars low quality. Inter-rater agreement for quality assessment was calculated using Cohen's kappa coefficient. Disagreements were resolved through consensus discussion.

Data Analysis

Statistical analyses were performed using Review Manager (RevMan) version 5.4 (The Nordic Cochrane Centre, Copenhagen, Denmark) and R software version 4.3.0 (R Foundation for Statistical Computing, Vienna, Austria) with the meta and metafor packages. For dichotomous outcomes (anemia correction), relative risks (RR) with 95% confidence intervals (CI) were calculated, while for continuous outcomes (hemoglobin changes), standardized mean differences (SMD) with 95% CI were computed. Random-effects models were employed as the primary analysis approach, accounting for anticipated clinical and methodological heterogeneity. Statistical heterogeneity was evaluated using Cochran's Q test (significance level p<0.10) and quantified using the I² statistic, where I² values of 25%, 50%, and 75% represented low, moderate, and high heterogeneity, respectively [18]. Publication bias was not assessed, as the number of studies was less than 10.

Results

**Figure 1:**
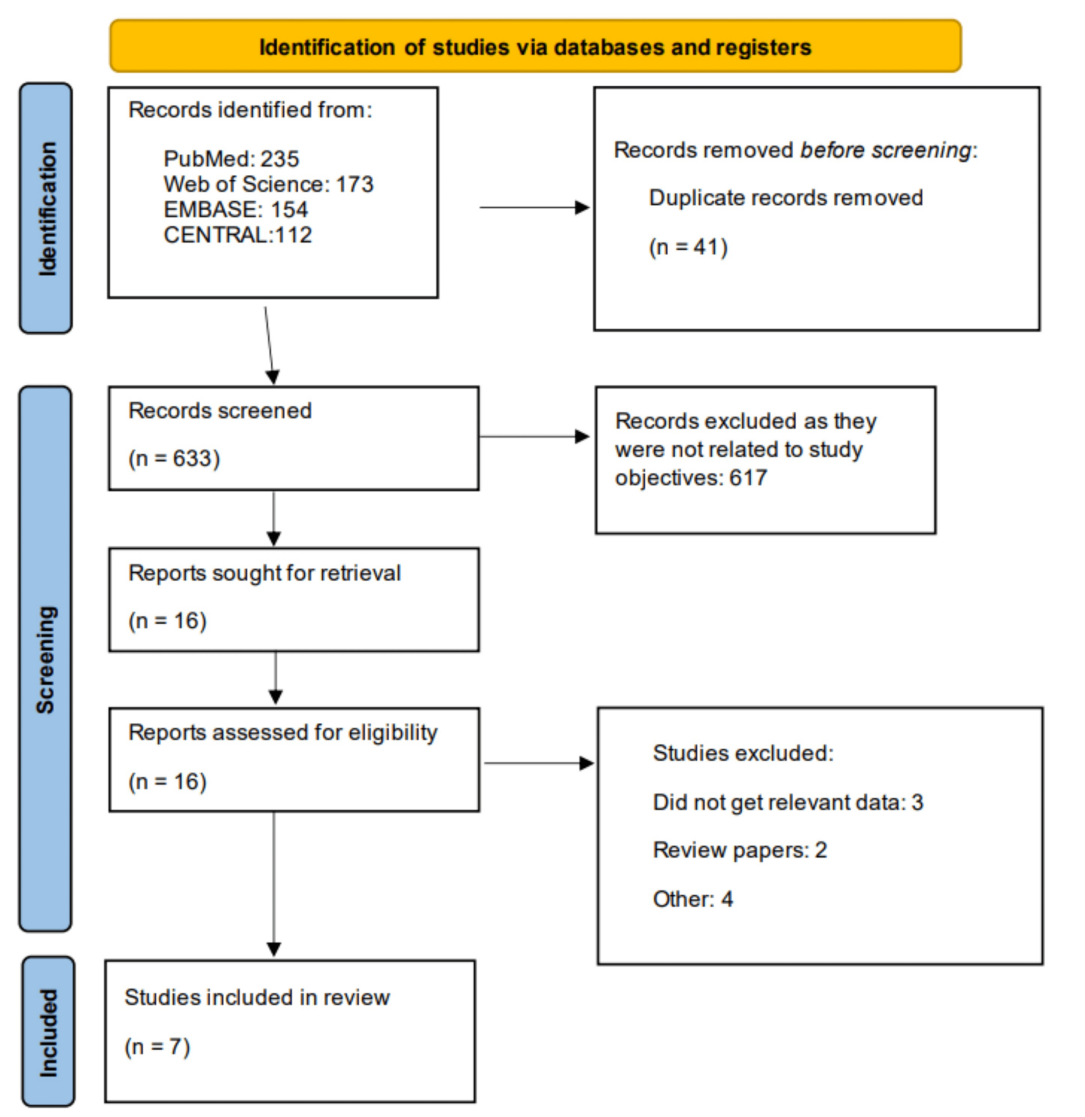
PRISMA flowchart (study selection process)

Figure 1 shows the PRISMA flowchart of the study selection process. Through database searching, we identified 674 studies. After removing duplicates, the remaining articles underwent initial screening, followed by a detailed full-text assessment for eligibility. Ultimately, seven studies comprising 15,540 participants were included in this analysis. Table 1 summarizes the characteristics of the included studies. These were conducted across diverse geographical regions, including single-country studies from India, Egypt, and Iraq, as well as large multinational trials spanning 9 to 21 countries. All studies evaluated dapagliflozin 10 mg once daily. In terms of study design, three were post-hoc analyses of RCTs, one was a single-center RCT, and two were observational studies. Additionally, one study was a pooled post-hoc analysis of 14 RCTs conducted before 2020, as their individual post-hoc results were not available. Given the importance of these data, this study was also included in the pooled analysis. Table 2 presents a quality assessment of included studies.

**Table 1:** Included studies’ characteristics RCT: Randomized-control trial; NR: Not reported

Author	Year	Study Design	Regions	Follow-up Duration	Sample Size	Dapagliflozin dose	Mean age	Heart Failure (n)	Males (n)	DIabetes (n)	Chronic kidney disease (n)	Anemia (n)
Bhaganagarapu et al. [14]	2025	RCT	India	6 Months	46	10 mg once daily	NR	46	28	46	16	46
Docherty et al. [10]	2021	RCT	20 Countries	18.2 Months	4691	10 mg once daily	66.3	4691	3598	1959	1905	1032
Elwaraky et al. [19]	2025	Cohort	Egypt	12 Months	255	10 mg once daily	64	42	135	255	255	175
Koshino et al. [20]	2023	RCT	9 Countries	6 Months	360	10 mg once daily	64.4	0	258	360	360	NR
Koshino et al. [21]	2023	RCT	21 Countries	28.8 Months	4304	10 mg once daily	61.8	465	2873	2906	4304	1716
Obead et al. [22]	2023	Cohort	Iraq	4 Months	100	10 mg once daily	63	100	67	69	100	NR
Steffanson et al. [23]	2020	Post-hoc analysis of 14 RCTs	Multi-national	At least 6 Months	5325	10 mg once daily	59.7	2290	3117	5325	1166	700

**Table 2:** Quality assessment of included studies For RCTs (RoB 2 domains): Domain 1: Bias arising from the randomization process; Domain 2: Bias due to deviations from intended interventions; Domain 3: Bias due to missing outcome data; Domain 4: Bias in measurement of outcome; Domain 5: Bias in selection of reported result For Observational Studies (NOS domains): Domain 1 Selection: Selection of study groups (maximum four stars); Domain 2 Comparability: Comparability of groups (maximum two stars); Domain 3 Outcome: Assessment of exposure/outcome (maximum three stars)

Study	Assessment Tool	Domain 1	Domain 2	Domain 3	Domain 4	Domain 5	Overall Quality
Randomized Controlled Trials (RoB 2)							
Bhaganagarapu et al., 2025 [14]	RoB 2	Low risk	Low risk	High risk	Low risk	High risk	High risk of bias
Docherty et al., 2021 [10]	RoB 2	Low risk	Low risk	Low risk	High risk	Low risk	Low risk of bias
Koshino et al., 2023 [20]	RoB 2	Low risk	Low risk	Low risk	Low risk	High risk	Low risk of bias
Koshino et al., 2023 [21]	RoB 2	Low risk	Low risk	Low risk	Low risk	High risk	Low risk of bias
Observational Studies (NOS)							
Elwaraky et al., 2025 [19]	NOS	3	2	2			Good
Obead et al., 2023 [22]	NOS	4	2	2			Good

Change in Hemoglobin

**Figure 2:**
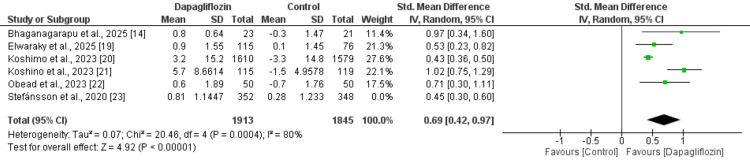
Comparison of change in hemoglobin between two groups References [14,19-23]

Six studies were included in the pooled analysis assessing the change in hemoglobin from baseline, and the results are presented in Figure 2. The analysis demonstrated that the increase in hemoglobin was significantly greater in participants receiving dapagliflozin compared with those in the control group (SMD: 0.62; 95% CI: 0.43 to 0.80). However, substantial heterogeneity was observed across the included studies (I² = 76%).

Incident of Anemia

**Figure 3:**
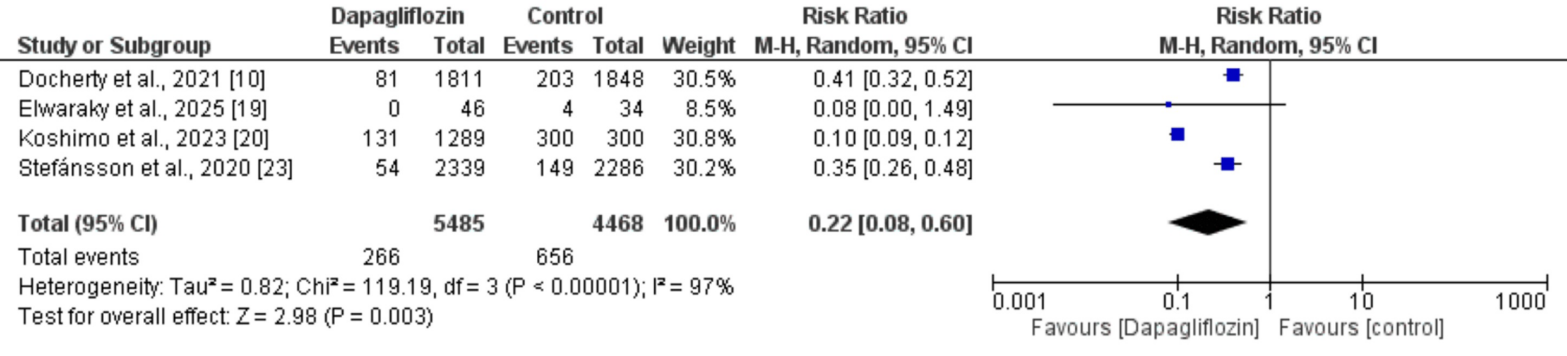
Comparison of anemia incidence between two groups References [10,19-20, 23]

Four studies evaluated the incidence of anemia, with results presented in Figure 3. The pooled analysis demonstrated that the risk of developing anemia was significantly lower among participants receiving dapagliflozin compared with those in the control group (RR: 0.22; 95% CI: 0.08-0.60). Nonetheless, considerable heterogeneity was observed across the studies (I² = 97%).

Correction of Anemia

**Figure 4:**
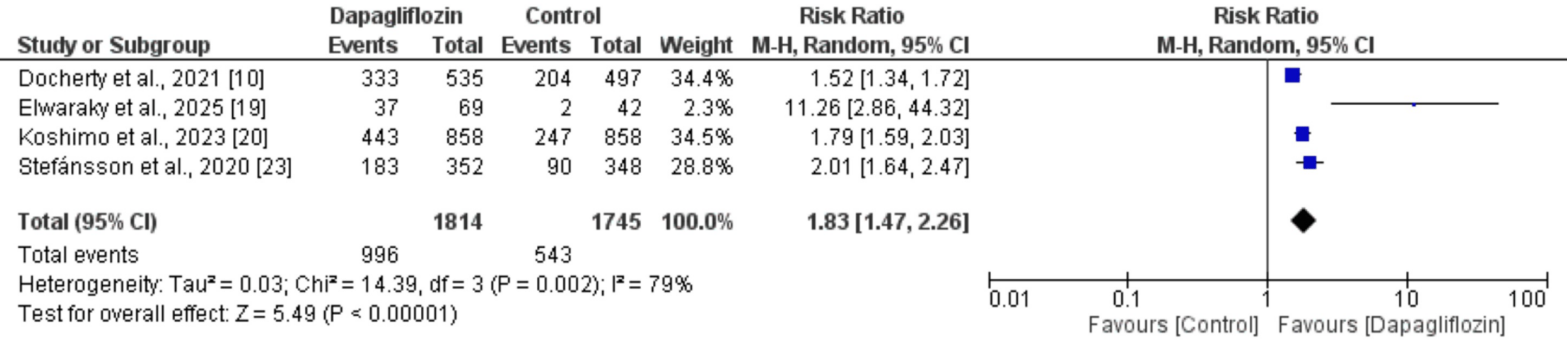
Comparison of anemia correction between two groups References [10,19-20, 23]

Four studies assessed anemia correction, and the findings are presented in Figure 4. The pooled analysis indicated that anemia correction was significantly more likely in participants receiving dapagliflozin compared with those in the control group (RR: 1.83; 95% CI: 1.47-2.26). However, substantial heterogeneity was observed among the study results (I² = 79%).

Subgroup Analysis

Subgroup analyses were performed by restricting to studies in which the entire study population had a specific condition (100% of participants). As shown in Table 3, dapagliflozin was associated with a significant increase in hemoglobin across all subgroups. The effect was most pronounced in studies enrolling patients with heart failure (SMD: 0.78; 95% CI: 0.44-1.12; I² = 0%), followed by those with chronic kidney disease (CKD) (SMD: 0.66; 95% CI: 0.36-0.95; I² = 44%) and diabetes (SMD: 0.45; 95% CI: 0.38-0.51; I² = 2%). Additionally, subgroup analysis of RCT and observational studies showed a positive effect of dapagliflozin on hemoglobin levels. 

**Table 3:** Subgroup analysis (change in hemoglobin) CKD: Chronic kidney disease; SMD: Standardized mean difference; CI: Confidence interval

Subgroup	SMD (95% CI)	I-Square
CKD	0.66 (0.36, 0.95)	44%
Heart Failure	0.78 (0.44, 1.12)	0%
Diabetes	0.45 (0.38, 0.51)	2%
Study Design (RCT)	0.64 (0.39, 0.88)	74%
Study Design (Observational)	0.59 (0.35, 0.83)	0%

Discussion

This systematic review and meta-analysis evaluated the impact of dapagliflozin. We observed that treatment with dapagliflozin led to a significant rise in hemoglobin levels compared with the control groups. Moreover, dapagliflozin use was associated with a higher likelihood of anemia correction and a reduced incidence of anemia. Consistent with our findings, a meta-analysis by Choi et al. demonstrated that SGLT2 inhibitors overall significantly increased hemoglobin levels compared with controls [9]. The elevation in hemoglobin seen with dapagliflozin is thought to contribute to renal and cardiovascular protection, primarily through enhanced tissue oxygen delivery [24]. Additionally, the diuretic properties of SGLT2 inhibitors, which decrease plasma volume, may partly account for this effect [25]. Another proposed mechanism is the stimulation of erythropoietin production, facilitated by reduced renal fibrosis and improved survival of erythropoietin-producing cells [26].

The anti-inflammatory properties of SGLT2 inhibitors may also contribute to anemia improvement. Chronic inflammation, prevalent in both heart failure and chronic kidney disease, suppresses erythropoiesis through multiple pathways, including hepcidin upregulation and direct bone marrow suppression [27]. By reducing inflammatory cytokines and oxidative stress, dapagliflozin may ameliorate anemia of chronic disease, a common finding in the studied populations [28]. Volume contraction effects, while initially proposed as the primary mechanism, likely play a secondary role. The sustained nature of hemoglobin improvements observed across studies, extending well beyond the initial diuretic phase, suggests that true erythropoietic stimulation rather than hemoconcentration underlies these benefits.

Our subgroup analysis revealed benefits in heart failure, CKD, and diabetes subjects. The multifactorial nature of heart failure-associated anemia creates multiple therapeutic targets for SGLT2 inhibitor intervention. Dapagliflozin's anti-inflammatory properties may directly counteract the cytokine-mediated suppression of erythropoiesis that characterizes anemia of chronic disease [28]. The substantial benefit observed in CKD patients is particularly clinically relevant given the inherent challenges in managing anemia in this population. Chronic kidney disease-associated anemia results primarily from decreased erythropoietin production as renal mass declines but is complicated by iron deficiency, chronic inflammation, and uremic toxins that directly suppress bone marrow function [29]. Traditional therapeutic approaches face significant limitations: erythropoiesis-stimulating agents, while effective, carry cardiovascular safety concerns, including increased risks of stroke, thromboembolism, and mortality when targeting higher hemoglobin levels [30].

The therapeutic advantage of dapagliflozin in chronic kidney disease extends beyond anemia management. Unlike traditional anemia treatments that address only hematological parameters, dapagliflozin simultaneously provides cardiovascular and renal protection through mechanisms including reduced intraglomerular pressure, decreased albuminuria, and improved endothelial function [31]. This comprehensive benefit profile is particularly valuable in chronic kidney disease patients, who face exponentially increasing cardiovascular risk as renal function declines [32]. The ability to address multiple therapeutic targets with a single agent represents a significant advancement in the management of this complex patient population.

The improvement in anemia parameters with dapagliflozin treatment has important clinical implications beyond hematological outcomes. Anemia correction is associated with improved quality of life, enhanced exercise tolerance, and potentially reduced cardiovascular risk in patients with chronic diseases [33]. The reduced incidence of new-onset anemia observed in our analysis (78% relative risk reduction) suggests a protective effect that may prevent the cascade of complications associated with progressive anemia. The clinical relevance of anemia improvement extends to healthcare resource utilization. Elwaraky et al. reported that erythropoiesis-stimulating agent (ESA) therapy initiation was significantly lower in dapagliflozin-treated patients (7.3% vs. 24.2%, p=0.002), suggesting potential cost savings and reduced treatment burden [5]. This finding has important implications for healthcare economics and patient quality of life.

Several areas warrant further investigation to optimize the clinical application of dapagliflozin's hematopoietic properties. Long-term studies are needed to establish the durability of anemia improvements and their impact on cardiovascular and renal outcomes. The relationship between anemia correction and dapagliflozin's established cardiovascular benefits requires clarification, as improved oxygen-carrying capacity may contribute to the drug's overall protective effects.

Study Limitations

Several important limitations must be acknowledged in interpreting these results. The relatively small number of included studies (n=7) limited our ability to perform comprehensive subgroup analyses and precluded assessment of publication bias using standard statistical tests, which typically require a minimum of 10 studies for reliable interpretation. The inclusion of both randomized controlled trials and observational studies, while providing broader real-world evidence, introduced methodological heterogeneity that may have influenced the pooled estimates. The substantial statistical heterogeneity observed across all primary analyses (I² = 76-97%) represents a significant limitation requiring cautious interpretation of pooled effect estimates. This heterogeneity likely reflects differences in study populations, baseline anemia prevalence and severity, treatment duration, outcome measurement methods, and definitions of anemia correction across studies. The inability to access individual patient data prevented more sophisticated analyses that might have explained sources of this heterogeneity. Furthermore, the diverse geographical locations and healthcare settings of the included studies, while enhancing external validity, may have introduced variability in standard care practices, anemia management protocols, and patient characteristics that could not be adequately controlled for in the analysis. The varying follow-up durations across studies also limited our ability to assess the temporal profile and sustainability of dapagliflozin's hematopoietic effects.

## Conclusions

This systematic review and meta-analysis provide compelling evidence that dapagliflozin significantly improves anemia outcomes across diverse patient populations. Treatment with dapagliflozin demonstrated consistent benefits in increasing hemoglobin levels, promoting anemia correction, and reducing anemia incidence compared to control groups. The therapeutic effects were most pronounced in heart failure patients, followed by those with chronic kidney disease and diabetes. These hematopoietic benefits likely result from multifactorial mechanisms, including enhanced erythropoietin production, reduced inflammation, and improved tissue oxygenation. The findings suggest that dapagliflozin's clinical utility extends beyond established cardiovascular and renal benefits to include meaningful anemia management. Given the substantial burden of anemia in chronic disease populations, these results support considering dapagliflozin as a valuable therapeutic option that simultaneously addresses multiple pathophysiological targets. Future long-term studies should investigate the durability of these effects and their impact on patient-centered outcomes.
